# Increase in Distant Stage Breast Cancer Incidence Rates in US Women Aged 25–49 Years, 2000–2011: The Stage Migration Hypothesis

**DOI:** 10.1155/2015/710106

**Published:** 2015-01-08

**Authors:** Anthony P. Polednak

**Affiliations:** Private Practice, 241 Stonecrest Drive, Bristol, CT 06010, USA

## Abstract

*Background.* Unexplained increases have been reported in incidence rates for breast cancer diagnosed at distant stage in younger U.S. women, using data from the Surveillance, Epidemiology and End Results (SEER) Program. *Methods.* This report focused on recent SEER trends (2000–2011) in age-standardized incidence rates of invasive breast cancer at ages 25–39 and 40–49 years and the hypothesis that stage migration may have resulted from advances in detecting distant metastases at diagnosis. *Results.* Increases in the rates for distant stage were roughly equal to decreases in the rates for the most advanced stage subgroups within regional stage; this was evident for estrogen receptor (ER) negative cancers, associated with poorer prognosis, but not for ER positive cancers. The 3-year relative survival rate increased over time for distant stage (especially in the ER positive subgroup) and regional stage but not for localized stage; these trends do not contradict the stage-migration hypothesis. *Conclusions.* Findings provide some support for stage migration as one explanation for the recent increase in incidence of distant stage breast cancer, but additional studies are needed using other databases.

## 1. Introduction

A statistically significant increase (1976–2009) has been reported in the age-standardized incidence rate (ASIR) for distant stage breast cancer diagnosed in women at age 25–39 years in the population covered by population-based cancer registries of the National Cancer Institute's Surveillance, Epidemiology, and End Results (SEER) Program [1]. The report raised concerns regarding increasing rates of aggressive breast cancer in young women [2, 3], especially because the increases were largest for age 25–39 years in the most recent time period examined (i.e., 2000–09) with a slight increase also at age 40–54 years [1]. Support for stage migration as an explanation for the long-term trends was regarded as limited [1], and the increase in distant stage incidence in young women is unexplained [4].

The present report focused on recent trends (2000–2011) in stage-specific ASIRs at age <50 years in relation to the hypothesis of stage migration, which could result from improvements in the detection of distant metastases [5], as shown for lung cancer [6]. If the prognosis of cases migrating to distant (metastatic) stage tends to be worse than most other patients classified as regional stage but better than most classified as distant, then spurious improvements may occur in survival rates for both stages. This is known as the Will Rogers phenomenon, as shown for lung cancer [6]. The present report included examination of recent trends in stage-specific survival rates for younger breast cancer cases in SEER registries.

## 2. Materials and Methods

SEER^*^Stat Version 8.1.5 software was used with the November 2013 SEER submission database that included cancers diagnosed in 2000–2011 for 18 SEER registries [7], which together cover about 28% of the US population [5, 8]. Invasive breast cancer was defined by International Classification of Diseases for Oncology Version 3 (ICD-O-3) site codes C500–C509, excluding ICD-O-3 Morphology (M) codes M9140 (Kaposi sarcoma) and M9590-9992 (mainly lymphomas) [7]. The focus was on women diagnosed at ages 25–39, an age group used in the previous report [1], and 40–49 years.

The variable labeled “summary stage 2000 (1988+)” [7] was used, as in most SEER publications (including survival rates) [5]. Summary stage is derived by converting data from two detailed SEER staging systems for diagnoses prior to 2004 versus 2004–2011 [7], including localized (i.e., confined to breast), regional (i.e., spread to adjacent lymph nodes or chest wall), and distant (i.e., distant sites such as bone and/or lymph nodes involved). Only 705 (2.1%) of all 33,704 invasive cancers diagnosed in 2000–2011 at age 25–39 years and 1858 (1.6%) of 115,035 at age 40–49 years had unknown summary stage. Inflammatory breast carcinomas that are regional by direct extension only, and cases with ipsilateral infraclavicular lymph node involvement only, are defined as regional in summary stage (versus distant in historic stage); any supraclavicular node involvement is distant stage in both schemes [9]. Ipsilateral infra- or supraclavicular node cases are stage III (regional) in the American Joint Committee on Cancer (AJCC) 6th edition TNM system (i.e., T for tumor size, N for node involvement, and M for distant metastasis) [10]. Thus, summary stage involves fewer distant but more regional cases and is closer to AJCC 6th edition, compared to historic stage.

Also used was the SEER breast-adjusted AJCC 6th stage (1988+) variable [7, 11] modified from the AJCC 6th edition; in merging codes from two different detailed SEER staging systems, for consistency, all distant lymph node involvements were coded as N3, rather than M1 (distant), in breast-adjusted stage [11]. For this study, however, breast-adjusted stage was used only to define subgroups within regional summary stage, which excludes distant metastatic sites. In breast-adjusted stage, however, infraclavicular lymph node involvement was coded as N3 only if other criteria were met (e.g., clinically apparent ipsilateral internal mammary nodes or pathologic finding of 10+ axillary nodes) [11]. Thus, breast-adjusted IIIC (i.e., any T, with N3) differs from IIIC in AJCC 6th edition. Other subgroups analyzed included stages IIIB (i.e., T4, N0–N2), IIIA (i.e., T0–T2, N2 or T3, N1-N2), and II (i.e., T0 or T1, N1, or T2, N0, or T2, N1) [10, 11].

Data were available in the SEER database for estrogen receptor (ER) and progesterone receptor (ER) tumor markers for 2000–2011, but not for the human epidermal growth factor 2 receptor (HER2) expression marker (available only for 2010-11) [7]. ER and PR status were coded in the database as positive (+), negative (−), borderline, and unknown [7].

ASIRs per 100,000 per year for women within ages 25–39 years and 40–49 years (using 5-year subgroups) directly standardized to the age distribution of the 2000 US population and 95% confidence limits (CL) were obtained using SEER^*^Stat [7]. Age-specific rates for older ages were obtained for comparison. The annual percent change (APC), estimated by fitting regression models (least squares) to the natural logarithm of each annual rate, *P* values (two-sided, with null hypothesis of APC = zero), and lower and upper 95% CL on APCs were obtained using SEER joinpoint regression program version 4.0.1 January 2013.

Using the SEER^*^Stat survival session (Ederer II actuarial method) [3, 7], 3-year relative survival rates (RSR) were adjusted (by age, gender, and race) for expected mortality in the general US population; 100% RSR indicates no survival disadvantage in patients versus the general population [3]. With follow-up on vital status through the end of 2011 [7], the 3-year RSR included diagnoses through 2008. CL (95%) on each RSR were estimated as ± [(standard error) × 2]. RSR analyses, by age at diagnosis group, included only persons diagnosed with invasive breast cancer as their first or only reportable tumor in the database and excluded cases ascertained in SEER only by death certificate (i.e., date of diagnosis unknown) or autopsy (i.e., with no survival after diagnosis) [7].

## 3. Results

**Figure 1:**
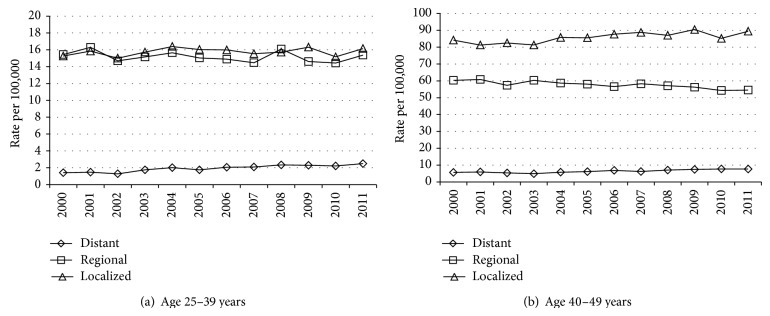
Age-standardized incidence rate for invasive breast cancer diagnosed at ages 25–39 years and 40–49 years in 2000–2011, SEER registries, by summary stage.

For age 25–39 years, the ASIR for distant summary stage increased with an APC of 5.4% (*P* < .001, Table 1); the higher ASIR for regional summary stage tended to decline (APC = −0.4%, Table 1) but fluctuated, with an increase from 2010 to 2011 (Figure 1(a)). The ASIR at age 25–39 years increased for localized summary stage (Table 1, Figure 1(a)); the ASIR declined for unknown summary stage from 2000 (ASIR = 1.1, *N* = 99) to 2005 (ASIR = 0.5, *N* = 39) but not thereafter (ASIR = 0.6, *N* = 45, in 2011) (data not shown).

For age 40–49 years, the ASIR for distant summary stage increased (APC = 3.6%, *P* < .001), whereas the ASIR for regional summary stage declined (Table 1) (APC = −0.9%, *P* < .001) (Figure 1(b)); the ASIR for unknown summary stage declined from 2000 (4.3, *N* = 262) to 2004 (1.8, *N* = 116) but not thereafter (2.1, *N* = 131, in 2011) (data not shown).

A previous study using distant historic stage reported a small positive APC for 2000–2009 for the ASIR in the broader age group 40–54 years [1].  Using distant historic stage the APC for 2000–2011 for the ASIR for the age group 40–49 years, however, was statistically significant (2.4%, CL = 1.3, 3.6%, *P* = .009) (data not tabulated).

Using distant summary stage, the APC (2000–2011) tended to decline with rising age—that is, 6.2% (CL = 3.2, 9.3%) at age 25–34, 5.0% (CL = 3.1, 8.9%) at 35–39 years, 4.6% (CL = 2.8, 6.3%) at 40–44 years, 3.0% (CL = 1.2, 4.8%) at 45–49, 1.6% (CL = 0.1, 3.0%) at 50–54 years, 2.1% (CL = 0.9, 3.2%) at 55–59 years, and 1.4% or less at older 5-year age groups through 75–79 years.

**Figure 2:**
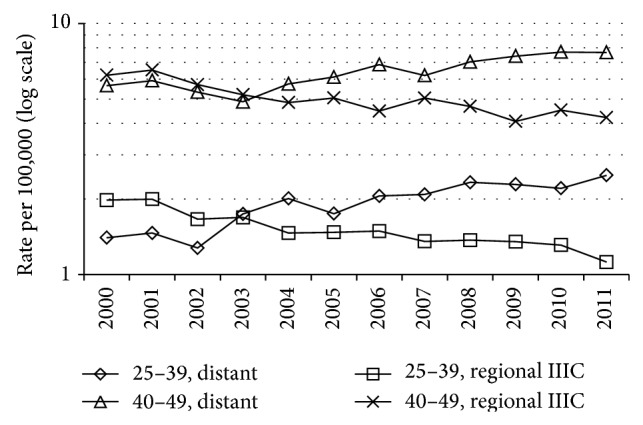
Age-standardized incidence rate for breast cancer diagnosed at ages 25–39 years and 40–49 years in 2000–2011: distant summary stage versus SEER breast-adjusted AJCC 6th stage IIIC within regional summary stage.

Within regional summary stage, the ASIR for age 25–39 years for breast-adjusted AJCC stage IIIC declined (APC = −4.5%, *P* < .001, Table 1) (Figure 2), with a smaller decline for IIIB and no decline for IIIA or IIA-IIB (Table 1); the remainder were 144 coded as stage III not otherwise specified (NOS), 111 as other, and 475 as unknown (including 44 cases in 2000 and 46 in 2011) (data not tabulated). Within regional summary stage, the ASIR for age 40–49 years declined for breast-adjusted AJCC stage IIIC (APC = −3.7%, *P* < .001, Table 1) (Figure 2), with a smaller decline for stage IIIB (Table 1); the remainder were 341 coded as III-NOS, 390 as other, and 1362 as unknown (including 88 cases in 2000 and 87 in 2011) (data not tabulated). Using historic (instead of summary) regional stage, statistically significantly negative APCs in rates were also obtained for IIIC at ages 25–39 (total *N* = 1315 cases) and 40–49 years (total *N* = 3278 cases).

The ASIR for all invasive breast cancers did not increase over time for age 25–39 years (APC = 0.1%, CL = −0.2, +0.4) or for age 40–49 years (APC = 0.1%, CL = −0.2, +0.4). The age-specific rate for all invasive cancers for age 50–54 years declined (APC = −1.1%, CL = −1.6, −0.6%, *P* = .001) but increased for distant summary stage (APC = 1.6%, CL = 0.1, 3.0%, *P* = .037) while declining for regional summary stage (APC = −2.0%, CL = −2.5, −1.5%, *P* < .001); the pattern was similar for age groups 55–59, 60–64, 65–69, and 70–74 years (data not shown).

In analyses by ER/PR markers, data for ages 25–39 and 40–49 were combined due to small samples of distant stage cancers within each age group. ER+/PR+ comprised 83,375 (57%) of all 146,176 cancers with known stage, with 33,103 ER−/PR− (23%), 11,540 (8%) ER+/PR−, 2,657 (2%) ER−/PR+, 13,012 (9%) unknown for both ER and PR, and 2,489 (2%) all other categories combined (comprised mainly of ER+/PR unknown, and ER and/or PR borderline). The ASIR increased for distant stage but declined for regional stage in the ER−/PR− subgroup, which also showed a decline for AJCC breast-adjusted IIIC within regional stage that was large enough to account for the increase in the distant stage ASIR (Table 2). The APC was negative for ER+/PR− cancers with breast-adjusted stage IIIC within regional stage but numbers were small. For ER+/PR+ cancers, however, ASIRs increased for regional and localized stages as well as for distant stage (Table 2). Stage-specific rates for unknown ER status declined, especially from 2003 to 2004 (with the advent of a new staging system), but were too small to explain the increase in distant stage ER+ rates or the absence of a post-2004 decline in breast-adjusted IIIC regional stage ER+ rates (data not tabulated).

The 3-year RSR for women aged 25–39 years at diagnosis increased substantially from 2000-01 to 2007-08 for both regional and distant summary stages, with only a slight increase for localized summary stage (Table 3). The trends were similar for women diagnosed at age 40–49 years (Table 3). The increases in 3-year RSR for distant and regional stages at age 25–49 years were greater for ER−/PR− cancers than for ER+/PR+ cancers (which had much higher RSR rates) (Table 3). Samples were too small for analysis of RSR for distant stage ER+/PR− cancers.

## 4. Discussion

Increases in distant summary stage ASIRs for ages 25–39 and 40–49 years were similar in magnitude to the declines for breast-adjusted AJCC IIIC stage within regional summary stage (Table 1, Figures 1 and 2), suggesting the hypothesis of stage migration. The decline in the rates for regional summary stage for age 40–49 years (and older ages) could have been affected by trends in breast cancer screening, but increases in mammography screening rates have not been reported since 2000 [12]. The greater increase in distant stage rates at ages <50 years versus older ages could reflect the tendency toward more aggressive cancers at younger ages [13], with a greater opportunity for an impact of technical advances in detection of distant metastases (versus older ages).

For lung cancer, a decline in diagnoses at stage III and an increase for stage IV coincided with increased use of positron emission tomography (PET) recorded in cancer registries in California [14].

For breast cancer, recent changes in staging include combining standard bone scans (scintigraphy) with MRI and computed tomography (CT) with ^18^fluorodeoxyglucose- (FDG-) PET scans which reportedly have higher sensitivity and specificity than conventional imaging in detecting distant metastases [15–17]. Bone and lung are the most common sites for distant metastases from breast cancer. Bone scans are sensitive in detecting osseous metastases, and MRI or PET-CT also may be considered for some cases with abnormal radionuclide uptake [18]. PET/CT examines the chest, bone, and abdomen in a single session, with a nonnegligible yield of evidence for occult distant metastases in patients with an initial diagnosis of clinical stage II or (especially) III cancer [19].

FDG-PET was described in a 2001 report as likely to become more widely used for staging [20] and in a 2003 report as “rapidly proliferating” (including insurance coverage by the Centers for Medicare and Medicaid Services in late 2002) [21]. FDG-PET is optional in some clinical guidelines [22] but may be especially useful for detecting metastases in patients diagnosed clinically as AJCC stage IIIC [23]. An American Society of Clinical Oncology report did not find evidence supporting use of PET, CT, and radionuclide bone scans for staging of patients with newly diagnosed early clinical stage (0, I or II) breast cancer, but these techniques were considered “appropriate” for stage III because of the higher likelihood of occult metastases in patients with clinical stage III or with inflammatory cancer [24].

A limitation of the SEER database used [7], however, is lack of information on the specific techniques used in staging, as well as on the specific metastatic site(s) involved. SEER-Medicare linkages have examined trends in imaging modalities for breast cancer patients, but data are limited to diagnoses at age 65+ years [25]; other administrative databases may be useful for studies of younger patients.

Other study limitations include the use of a summary staging scheme that involves merging of codes from two different detailed staging systems for pre-2004 versus 2004+ diagnoses; however, the trends continued after 2004 (Figures 1 and 2). Also, the database used [7] did not include adjustment for delayed reporting of incident cases; however, such adjustment had little or no impact on APCs for ASIRs in 1992–2010 for all invasive breast cancer in SEER [5, 8].

 The evidence for stage migration in ER− but not ER+ cancers (Table 2) may be related to the probability of distant metastases. ER negative cancers, more common at younger ages, have distinct morphological features at diagnosis, including larger size and higher tumor grade [26], and the higher risk of recurrences in the early years after diagnosis suggests the presence of dormant micrometastases at diagnosis [27]. Hence, improvements over time in detection of clinically occult metastases at initial diagnosis would be more relevant to patients with ER− than ER+ cancer at diagnosis.

For ER+ cancers at age 25–49 years temporal increases in ASIRs were evident for each stage (although largest for distant stage), with only a slight decline for breast-adjusted stage IIIC within regional stage (Table 2). A previous study [1] reported trends by ER/PR status in young women only for distant stage cancers. Analyses of SEER data on overall (not stage-specific) incidence rates have shown increases for ER+ versus declines for ER− breast cancers (including age 30–49 years), and these divergent trends are unexplained [28]; the limitations of hormone-receptor data from cancer registries must be acknowledged, including missing data and temporal changes in sensitivity of tests [28].

These findings on trends in stage-specific incidence rates within the ER+/PR+ subgroup (Table 2), if confirmed, suggest that the stage migration hypothesis is not the only potential explanation for the recent increase (2000–2011) in overall distant stage incidence rates at age <50 years. Certain risk factors differ by ER/PR status in women <50 years of age at diagnosis (e.g., nulliparity and older age at first childbirth may be stronger risk factors for ER+/PR+ versus ER−/PR− cancers) [29] and these risk factors should be examined in future studies using other databases.

Three hypotheses involving temporal changes in potential risk factors for breast cancer have been proposed for the increase in distant stage cancer rates at age <50 years, but the fact that cancer registries do not routinely collect data on risk factors [1, 4, 30] makes such hypotheses speculative. Johnson et al. [4] discussed these three hypotheses: folate supplementation of grain products which became mandatory in 1998; the introduction of vaccines preventing certain childhood viral infections that might conceivably have protected against cancer development in later life; and increasing age at first pregnancy in US women. These hypotheses, however, did not appear to explain the specific age group (i.e., younger women) and stage category (i.e., metastatic) showing the increasing breast cancer incidence rates [4].

The temporal trend toward “advanced age at first full term pregnancy” among US women has probably resulted in increasing numbers of pregnancy-associated breast cancers (i.e., variously defined as diagnosed during pregnancy and within either a year or a few years postpartum) that may have a tendency toward late stage at diagnosis [30]. A response to this hypothesis [4], however, noted that distant stage incidence rates in SEER showed the largest temporal increase (1992–2009) for women aged 25–34 years [1], and the magnitude of the increase (APC) declined progressively with rising age [1, 4] as also found in the present study of rates for 2000–2011. In contrast, an increase in US women having their first childbirth at age 35+ (predominantly 35–44) years had the largest impact on the rising average age at first childbirth in US women over time, and this trend was greater during the 1970s and 1980s than in later years [31]. Clearly, studies are needed using databases other than SEER [1, 4]. Linkages of cancer registries with birth databases, for example, could be used to examine temporal trends in pregnancy-associated breast cancers, as done in Australia [32], but apparently studies have not reported trends for these breast cancers by stage at diagnosis and tumor markers.

The temporal increase in 3-year RSR for age 25–49 years within both regional and distant summary stages but not localized stage, especially within the ER−/PR− subgroup (Table 3), does not contradict the hypothesis of the Will Rogers phenomenon. Studies are needed, however, that include data on systemic therapies (endocrine therapy and chemotherapy) which were not included in the database [7] because routinely collected SEER data are incomplete. Limited studies on temporal trends in survival among patients with an initial (de novo) diagnosis of metastatic breast cancer have included data on systemic therapies but suggest modest increases in survival that may be due in part to improvements in endocrine therapy and the recent introduction of trastuzumab for certain subgroups of patients [33].

## 5. Conclusions

Findings provide some support for the hypothesis of stage migration as one potential factor in the increase in distant stage breast cancer incidence rates at age <50 years, mainly for ER negative cancers. Future studies should involve databases that can document the specific staging techniques used by year of diagnosis but also should consider alternative hypotheses involving temporal changes in risk factors for ER+ cancers (especially those diagnosed at distant stage). Studies should assess the impact of temporal advances in systemic therapies on trends in stage-specific survival rates for subgroups defined by tumor markers (ER, PR, and HER2) in addressing the hypothesis of the Will Rogers phenomenon.

## Figures and Tables

**Table 1:** Age-standardized incidence rate for women aged 25–39 years and 40–49 years with invasive breast cancer in SEER registries: annual percent change (APC) by stage at diagnosis^a,b^.

Stage	2000		2011		2000–2011		
	Number	Rate (CL)	Number	Rate (CL)	Number	Rate (CL)	APC, % (CL)
Age 25–39 years							
Distant	123	1.4 (1.2, 1.7)	204	2.5 (2.2, 2.9)	1935	1.9 (1.8, 2.0)	5.4 (3.7, 7.2)^*^
Regional	1356	15.4 (14.6, 16.3)	1249	15.4 (14.5, 16.3)	15,251	15.2 (14.9, 15.4)	−0.4 (−1.1, +0.3)
IIIC^b^	174	2.0 (1.7, 2.3)	94	1.1 (0.9, 1.4)	1541	1.5 (1.5, 1.6)	−4.5 (−5.5, −3.4)^*^
IIIB	88	1.0 (0.8, 1.2)	67	0.8 (0.6, 1.1)	911	0.9 (0.8, 1.0)	−0.9 (−2.3, +0.5)
IIIA	324	3.7 (3.3, 4.1)	324	4.0 (3.6, 4.4)	3773	3.8 (3.6, 3.9)	0.5 (−0.9, +2.1)
IIA, IIB	693	7.9 (7.3, 8.5)	704	8.7 (8.1, 9.4)	8296	8.3 (8.1, 8.4)	0.2 (−0.6, +0.9)
Localized	1338	15.3 (14.5, 16.1)	1309	16.1 (15.3, 17.1)	15,813	15.8 (15.5, 16.0)	0.2 (−0.3, +0.8)

Age 40–49 years							
Distant	341	5.7 (5.1, 6.3)	475	7.7 (7.0, 8.4)	4817	6.4 (6.2, 6.6)	3.6 (2.2, 5.1)^*^
Regional	3634	60.3 (58.3, 62.3)	3396	54.5 (52.7, 56.4)	43,479	57.7 (57.1, 58.2)	−0.9 (−1.2, −0.5)^*^
IIIC^b^	375	6.2 (5.6, 6.9)	263	4.2 (3.7, 4.8)	3795	5.0 (4.9, 5.2)	−3.7 (−4.9, −2.5)^*^
IIIB	206	3.4 (3.0, 3.9)	168	2.7 (2.3, 3.1)	2361	3.1 (3.0, 3.3)	−1.4 (−2.9, +0.2)
IIIA	809	13.4 (12.5, 14.4)	840	13.5 (12.6, 14.5)	9916	13.2 (12.9, 13.4)	0.0 (−0.6, +0.6)
IIA, IIB	2083	34.6 (33.1, 36.1)	2015	32.3 (30.9, 33.8)	25,314	33.6 (33.2, 34.0)	−0.6 (−1.1, −0.1)^*^
Localized	5078	84.2 (81.9, 86.5)	5603	89.4 (87.1, 91.8)	64,881	85.8 (85.1, 86.4)	0.8 (0.3, 1.2)^*^

^a^Summary stage 2000 (1988+) [7] (see text).

^b^SEER breast-adjusted AJCC 6th edition stages IIIC, IIIB, and IIA-B, within regional summary stage cancers [11] (see text).

CL: confidence limits (95%), lower and upper, on rate or on APC.

SEER: Surveillance, Epidemiology, and End Results Program of the National Cancer Institute.

^*^CL do not include zero.

**Table 2:** Trend in age-standardized incidence rate for women aged 25–49 years with invasive breast cancer in SEER registries, by estrogen (ER) and progesterone (PR) hormone-receptor status category: annual percent change (APC) by stage at diagnosis^a,b^.

Stage	2000		2011		2000–2011
	Number	Rate (CL)	Number	Rate (CL)	APC, % (CL)
ER negative/PR negative					
Distant	124	0.8 (0.7, 1.0)	178	1.2 (1.0, 1.4)	4.7 (2.4, 7.0)^*^
Regional	1177	8.0 (7.6, 8.5)	968	6.6 (6.2, 7.1)	−1.6 (−2.9, −2.0)^*^
IIIC^b^	165	1.1 (1.0, 1.3)	97	0.7 (0.5, 0.8)	−3.6 (−5.2, −2.1)^*^
Localized	1390	9.5 (9.0, 10.0)	1380	9.3 (8.9, 9.9)	−0.3 (−1.6, +1.0)

ER positive/PR negative					
Distant	37	0.3 (0.2, 0.3)	86	0.6 (0.5, 0.7)	8.0 (5.5, 10.5)^*^
Regional	373	2.5 (2.3, 2.8)	444	3.1 (2.8, 3.4)	0.8 (−0.5, +2.2)
IIIC^b^	47	0.3 (0.2, 0.4)	37	0.3 (0.2, 0.4)	−2.4 (−4.4, −0.3)^*^
Localized	414	2.8 (2.6, 3.1)	537	3.6 (3.3, 3.9)	2.6 (1.6, 3.6)^*^

ER positive/PR positive					
Distant	147	1.0 (0.8, 1.2)	361	2.5 (2.2, 2.7)	9.0 (7.6, 10.4)^*^
Regional	2387	16.3 (15.7, 17.0)	3014	20.5 (19.8, 21.2)	2.3 (1.8, 2.8)^*^
IIIC^b^	221	1.5 (1.3, 1.7)	212	1.4 (1.2, 1.6)	−1.0 (−2.6, +0.6)
Localized	3052	20.9 (20.2, 21.7)	4567	30.8 (29.9, 31.7)	4.0 (3.1, 4.8)^*^

^a^Summary stage 2000 (1988+) [7] (see text).

^b^SEER breast-adjusted AJCC 6th edition stage IIIC, within regional summary stage cancers [11] (see text and Table 1).

CL: confidence limits (95%), lower and upper, on rate or on APC.

SEER: Surveillance, Epidemiology, and End Results Program of the National Cancer Institute.

^*^CL do not include zero.

**Table 3:** Three-year relative survival rate (RSR) for women diagnosed at age <50 years with invasive breast cancer in SEER registries for selected years of diagnosis by summary stage at diagnosis^a^.

Years	Distant stage		Regional stage		Localized stage	
	Number^b^	RSR (CL)	Number^b^	RSR (CL)	Number^b^	RSR (CL)
Age 25–39 years						
2000-01	227	30.2 (24.0, 36.4)	2634	80.8 (79.2, 82.4)	2509	95.1 (94.1, 96.1)
2007-08	335	45.4 (39.6, 51.2)^*^	2418	87.2 (85.8, 88.6)^*^	2355	95.7 (94.7, 96.7)

Age 40–49 years						
2000-01	622	32.7 (28.9, 36.5)	6820	86.8 (86.0, 87.6)	9111	97.3 (96.9, 97.7)
2007-08	747	41.0 (37.2, 44.8)^*^	6737	90.2 (89.4, 91.0)^*^	9980	97.8 (97.4, 98.2)

Major ER/PR subgroups, age 25–49 years						
ER positive/PR positive^c^						
2000-01	287	50.1 (44.1, 56.1)	4602	93.2 (92.4, 94.0)	5702	98.9 (98.5, 99.3)
2007-08	499	56.6 (51.8, 61.4)	5635	95.8 (95.2, 96.4)^*^	7864	99.2 (98.8, 99.6)
ER negative/PR negative^c^						
2000-01	214	15.3 (10.3, 20.3)	2208	69.7 (67.7, 71.7)	2507	92.2 (90.0, 93.4)
2007-08	326	27.4 (22.2, 32.6)^*^	2223	75.4 (73.4, 77.4)^*^	2629	92.9 (91.7, 94.1)

^a^SEER summary stage 2000 (see text) [3, 7, 8].

^b^Number of cases at start of follow-up, after exclusion of those with breast cancer as other than their first or only reportable tumor in the SEER database, and breast cancers ascertained by death certificate or autopsy only (see text). With follow-up through December 2011 [7], all cases diagnosed in 2007-08 had the potential to survive at least 3 years after diagnosis (see text).

^c^Estrogen receptor (ER)/progesterone receptor (PR) (see Table 2 and text). Other ER/PR categories involved small numbers (see text).

CL: confidence limits, lower and upper (95%).

SEER: Surveillance, Epidemiology, and End Results Program of the National Cancer Institute.

^*^CL for RSR for this period do not overlap with CL for 2000-01.
